# Supplementary material for: Human-Specific Regulation of Neurotrophic Factors MANF and CDNF by microRNAs
Source: Int J Mol Sci. 2021 Sep 7;22(18):9691. doi: 10.3390/ijms22189691 (PMC8466963; doi:10.3390/ijms22189691)

Supplementary Materials

Table S1. List of miRNAs used in current study with their sequences, used prediction tools and conservation status.

Target gene	microRNA	Sequence	Tools	Conservation (based on TargetScan)
hMANF	hsa-miR-141-5p	CAUCUUCCAGUACAGUGUUGGA	D	N/A
	hsa-miR-144-3p	UACAGUAUAGAUGAUGUACU	M	N/A
	hsa-miR-544a	AUUCUGCAUUUUUAGCAAGUUC	M	N/A
	hsa-miR-338-3p	UCCAGCAUCAGUGAUUUUGUUG	M, T	Conserved
hCDNF	hsa-miR-134-5p	UGUGACUGGUUGACCAGAGGGG	M, T, D	Poorly conserved
	hsa-miR-141-5p	CAUCUUCCAGUACAGUGUUGGA	D, T	Poorly conserved
	hsa-miR-190a-5p	UGAUAUGUUUGAUAUAUUAGGU	M, T	Poorly conserved
	hsa-miR-382-5p	GAAGUUGUUCGUGGUGGAUUCG	M, T, D	Poorly conserved
	hsa-miR-539	GGAGAAAUUAUCCUUGGUGUGU	M, T, D	Poorly conserved
	hsa-miR-599	GUUGUGUCAGUUUAUCAAAC	M, T, D	Poorly conserved

M – miRanda; T – TargetScan; D – Diana Tools microT-CDS

Table S2. List of miRNA mimics used in this study

miRNA mimic	miRIDIAN catalog number
hsa-miR-134-5p	C-300628-05-0002
hsa-miR-141-5p	C-301056-01-0002
hsa-miR-144-3p	C-300612-05-0002
hsa-miR-190a-5p	C-300639-03-0002
hsa-miR-338-3p	C-300706-05-0002
hsa-miR-382-5p	C-300691-03-0002
hsa-miR-539-5p	C-300859-01-0002
hsa-miR-544	C-300860-03-0002
hsa-miR-599	C-300923-01-0002
NC (Scrb) mimic	CN-001000-01-05

Table S3. List of PCR primers used for amplification of 3′UTRs of MANF and CDNF (both human and mouse).

PCR primers	Primer sequences (F: forward; R: reverse)
Human MANF 3′UTR	F 5′ TCGCTCGAGTCTGCTCAATCTCTGTTGCAC<br>R: 5′ AGAGCGGCCGCAGGAGCCTCCTGGGGCA
Human CDNF 3′UTR	F: 5′ TCGCTCGAGTCTCCAATGCCAGCACATTTGT<br>R: 5′ AGAGCGGCCGCCCAAGTTGCCTATGAGTGTTTTTA
Mouse MANF fragment	F: 5′ TGCCTAAATACGCCCCCAAG<br>R: 5′ GTTTCGCTCGCTGCCTATCA
Mouse MANF 3′UTR	F: 5′ TAGGCGATCGCTCGAGTCTGCCCAATTCCTGCTGCACCTG<br>R: 5′ TTGCGGCCAGCGGCCGCCTCGGGGCCACGCCC
Mouse CDNF 3′UTR	F: 5′ TAGGCGATCGCTCGAGCCCCGTCGGCTGTCAG<br>R: 5′ TTGCGGCCAGCGGCCGCAATGTATATTTATTATCTGAATTTAAGTTATTC TCTTGCGGCCAGCGGCC

Table S4. List of PCR primers used for site-directed mutagenesis.

	miRNA, which binding site to be mutated	Primer sequences (F: forward; R: reverse)
hCDNF	miR-134	F: 5′-TTCATTCCTTACAacCACATATCAGACAAG<br>R: 5′-AAAGACCTTGAGCTTGGTTCAATTAACAAT
	miR-141	F: 5′-AAGTTTTTGATGGggGATTAAAGCATAT<br>R: 5′-GTCTGATATGTGACTGTAAGGAATGAAAAA
	miR-190a	F: 5′-CCTTACAGTCACAcgTCAGACAAGTT<br>R: 5′-AATGAAAAAGACCTTGAGCTTGGTTC
	miR-382	F: 5′-TATCTAATGCACggCTTCAATCCCAAATAC<br>R: 5′-AGCTGACTGTTGTGTCTTTTGTTTTAGTCC
hMANF	miR-144	F: 5′-TCCTGACAATAagGTATCAGATGTGA<br>R: 5′-GCCCACTTAAAAAATAAATTACAAAAAGG
	miR-544	F: 5′-TAGCTGTCCTTGCtcAATTATAGTGAA<br>R: 5′-GAAATTTTTTTCTTTAATAAAAAAATCCC

Table S5. List of PCR primers used for RT-qPCR analysis of ER stress markers expression.

PCR primers	Primer sequences (F: forward; R: reverse)
Human BiP	F 5′ ACCCTGACTCGGGCCAAAT<br>R: 5′ AGACCGGAACAGATCCATGTTG
Human ATF6	F: 5′ GGATGAAGTTGTGTCAGAGAACC<br>R: 5′ GACAACTCTTCGCTTTGGACTAG
Human CHOP	F: 5′ CCAGCAGAGGTCACAAGCA<br>R: 5′ TGACTGGAATCTGGAGAGTGAG
Human XBP1s	F: 5′ GAGTCCGCAGCAGGTG<br>R: 5′ CCGCCAGAATCCATGGG
Human GAPDH	F: 5′ TTGAGGTCAATGAAGGGGTC<br>R: 5′ GAAGGTGAAGGTCGGAGTCA

Supplementary Figure S1. miRNAs predicted to target 3'UTRs of mouse **A**) CDNF and **B**) MANF. Only miRNAs, included in the current study, are shown. Alignment is adapted from TargetScan and miRanda.

A

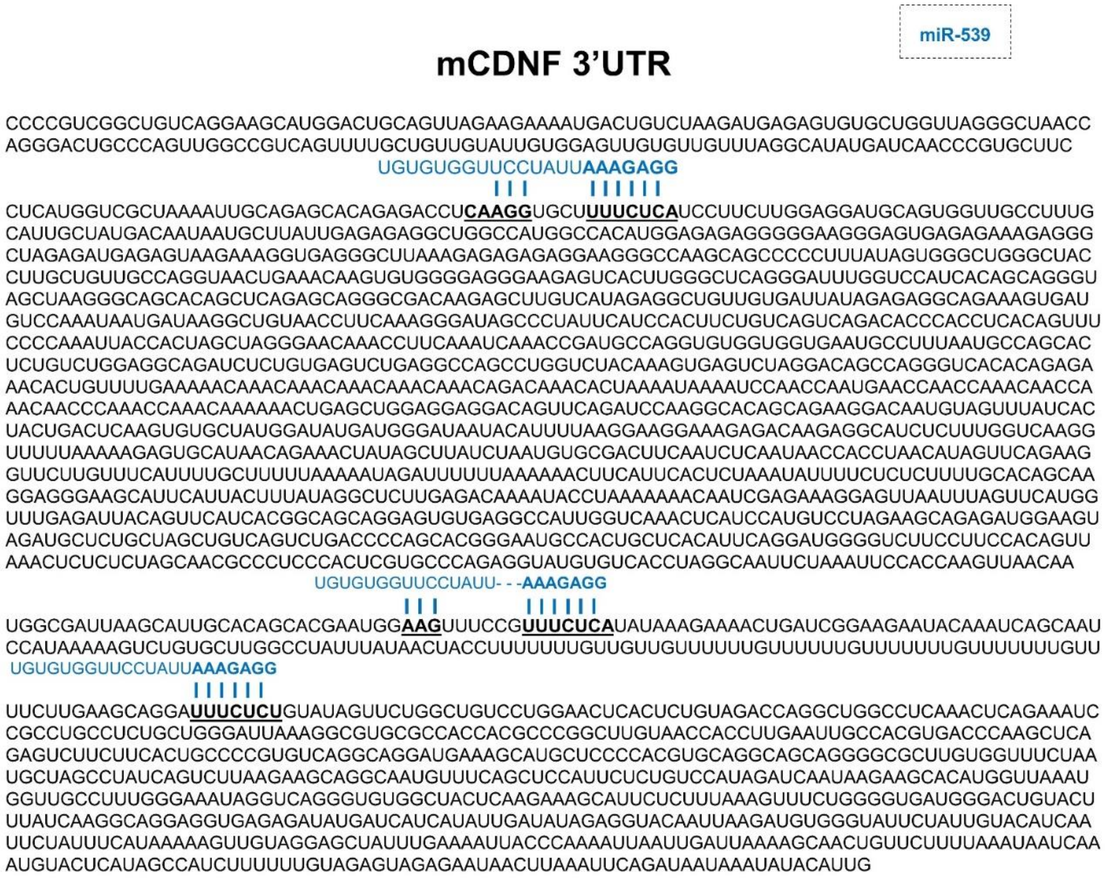

B

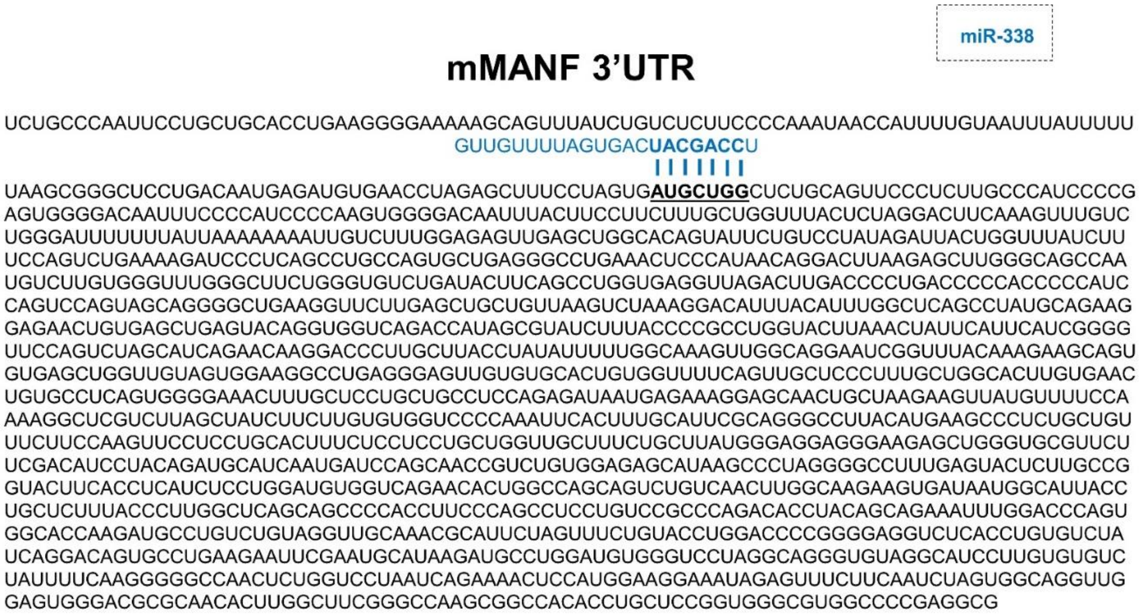

Supplement: Supplementary file 1 [file ijms-22-09691-s001.zip › ijms-1288426-supplementary.pdf]
